# Evaluating the use of pharmacological stress agents during single-photon emission computed tomography myocardial perfusion imaging tests after inadequate exercise stress test

**DOI:** 10.1007/s12350-021-02546-5

**Published:** 2021-03-11

**Authors:** Hongbo Yang, Elizabeth Faust, Emily Gao, Sakshi Sethi, Therese M. Kitt, Rita M. Kristy, James R. Spalding, Yanqing Xu

**Affiliations:** 1 Analysis Group, Boston, MA USA; 2 Astellas Pharma Global Development, Northbrook, IL USA

**Keywords:** SPECT, MPI, Exercise testing

## Abstract

**Background:**

Past clinical trial findings suggest that the availability of regadenoson in a nuclear imaging center may affect real-world center practices related to the transition of patients from an inadequate exercise stress test (EST) to a pharmacological stress agent (PSA).

**Methods and Results:**

This was a cross-sectional study using one-on-one telephone interviews with nuclear imaging center staff to facilitate survey development, followed by an online survey to evaluate patterns and processes around use of PSAs during single-photon emission computed tomography myocardial perfusion imaging (SPECT-MPI) in patients with inadequate ESTs. Of the 50 participants, 35 (70%) used only regadenoson, 3 (6%) only adenosine, 3 (6%) regadenoson and adenosine, 7 (14%) regadenoson and dipyridamole, and 2 (4%) all 3 agents for converting patients from an inadequate EST to a PSA. Nearly all centers (94%) used protocols to guide conversions. Of 12 centers using > 1 PSA, 11 reported regadenoson to be the most preferred PSA. Total staff time required from PSA transition to post-test monitoring was shortest for regadenoson.

**Conclusions:**

Compared to adenosine and dipyridamole, regadenoson is preferred by nuclear imaging center staff and associated with operational efficiencies after inadequate EST in real-world practice SPECT-MPI.

**Supplementary Information:**

The online version contains supplementary material available at 10.1007/s12350-021-02546-5.

## Introduction

Single-photon emission computed tomography myocardial perfusion imaging (SPECT-MPI) is a common non-invasive form of cardiac testing, with imaging performed at rest before and after exercise. Patients who are unable to exercise to an adequate workload are considered to have an inadequate exercise stress test (EST); a pharmacological stress test can be used to increase myocardial perfusion and induce flow heterogeneity in those with obstructive coronary artery disease.^1^,^2^ There are a variety of pharmacological stress agents (PSAs) available, including regadenoson, adenosine, dipyridamole, and dobutamine. Regadenoson, adenosine, and dipyridamole are cardiac vasodilators, which dilate coronary vessels to increase blood velocity and flow rate in normal vessels and cause less of a response in stenotic vessels; they are the preferred agents for MPI.^2^ In clinical practice, the choice of agent depends on patient characteristics, the stress imaging techniques being performed, and provider preference.

Regadenoson, administered intravenously via a pre-filled syringe,^3^ is one of the most widely used PSAs.^4^ The EXERRT (EXErcise to Regadenoson in Recovery Trial) phase 3 trial showed that administration of regadenoson could occur during recovery from the inadequate EST at the same visit, rather than in a follow-up visit.^5^ These clinical trial findings suggest that the availability of regadenoson in a nuclear imaging center may affect real-world center practices related to the transition of patients from an inadequate EST to a PSA.

The objective of the current study was to evaluate how PSAs are currently used for patients during SPECT-MPI assessments following an inadequate EST and, more specifically, to (1) describe the protocol/practice implemented in nuclear imaging centers to convert patients from an inadequate EST to a PSA, and (2) assess resource use and time associated with the use of regadenoson compared to other PSAs.

## Methods

### Study Design

This was a cross-sectional study utilizing an online survey conducted between June and July 2019 to evaluate the use of PSAs during SPECT-MPI tests following an inadequate EST (the survey is 27 pages and is therefore not included). One-on-one telephone interviews with staff from nuclear imaging centers were conducted to facilitate survey development. The interviews were conducted with 6 staff members from different United States (US) nuclear imaging centers: 3 nuclear technicians/technologists, 2 physicians, and 1 manager. The development of the web-based questionnaire was refined according to feedback obtained during interviews. No patients or patient records were involved in the one-on-one interviews or the online survey phases of this study.

Study materials, including the protocol and survey, were submitted to the New England Institutional Review Board for approval. To prepare for survey fielding, a pilot test with 2 respondents was conducted to ensure the logic and clarity of the questionnaire. During each pilot test, a moderator went through the questionnaire with the respondent and documented and addressed any questions or concerns regarding the questionnaire. The questionnaire was revised, as needed.

After the pilot test, a soft launch of the survey was conducted with about 10% of the target population. The data were reviewed to confirm that data quality met expectations. Following the soft launch, the online survey was fully launched, and data were collected via an online portal. The data were deidentified so they contained no information related to the identity of the participants.

### Participants

Survey respondents were selected from 50 different US-based nuclear imaging centers that provide outpatient SPECT-MPI testing so that no more than 1 staff member per center participated. Centers that met the inclusion criteria were included, regardless of whether the center had a protocol in place on the use of PSAs; however, sites were not selected based on use of a specific PSA. Interviewees were recruited by MedAxiom, a membership community that serves and supports more than one-third of all cardiology programs and practices across the US. MedAxiom used industry expertise to identify participants, which could include nuclear technicians, nurses, physician leaders (e.g., cardiologists), and imaging center managers/directors, with sufficient familiarity with the SPECT-MPI test procedure and use of PSAs. Nuclear imaging center staff were excluded from participation if they did not speak English or if they worked at the same nuclear imaging center as other respondents or at centers with PSA-first or PSA-only protocols (i.e., centers that do not conduct ESTs).

### Survey Instrument

The survey included a brief introduction, screening questions to confirm nuclear imaging center staff eligibility, and questions regarding nuclear imaging center and staff characteristics (e.g., demographics, provider setting, experience with SPECT-MPI test and PSAs, role and responsibilities). Additionally, respondents were asked about their experience with their center’s protocol/practice for conversion of an inadequate EST to a PSA during SPECT-MPI tests. The type and frequency of resources used, including staff time and laboratory resources, to conduct the tests were assessed.

### Analysis

As a cross-sectional study, no statistical inference or parameter estimation was required. The primary endpoints included (1) factors related to the procedure/practice in nuclear imaging centers to convert patients from an inadequate EST to a PSA, and (2) the type and frequency of resources used, including staff time and laboratory resources, during SPECT-MPI tests for patients who transition from an inadequate EST to a PSA.

Only data from nuclear imaging center staff who completed the entire survey were used for analyses. Characteristics of nuclear imaging center staff who completed the survey were summarized descriptively, as were data collected with regard to the current protocol/practices related to conversion from inadequate EST to PSA during SPECT-MPI tests and resource use and other factors related to PSA use. Continuous variables were summarized by mean and standard deviation, while categorical variables were summarized using frequency count and percentage for each question in the survey.

## Results

### Nuclear Imaging Staff and Center Characteristics

Of the 105 respondents, 50 completed the survey and were included in the analysis. Among the 55 respondents who did not complete the survey, 46 initiated but terminated the survey before completion for unknown reasons, 6 indicated they did not work closely with patients undergoing SPECT-MPI, 2 indicated they worked at centers that only did de novo pharmacological stress tests, and 1 did not initiate completion of the survey.

Characteristics of the nuclear imaging staff and centers are summarized in Table 1. More than half of the respondents (56%) were nuclear technicians/technologists, and the most common types of centers were hospital-affiliated diagnostic imaging centers (48%) or academic or teaching hospitals (18%). Most centers (94%) used protocols to convert patients from an inadequate EST to a PSA.

**Table 1:** Nuclear imaging staff and center characteristics

Characteristic	N = 50
Nuclear imaging center staff	
Years at current center, mean (SD)	12.3 (7.8)
Current role, n (%)	
Nuclear technician/technologist	28 (56.0)
Nurse	5 (10.0)
Physician	2 (4.0)
Nurse practitioner/physician assistant	6 (12.0)
Manager/Director	6 (12.0)
Other^a^	3 (6.0)
Years in current role, mean (SD)	18.7 (9.2)
Nuclear imaging center	
Setting, n (%)	
Non-academic, non-teaching hospital	4 (8.0)
Academic or teaching hospital	9 (18.0)
Diagnostic imaging center affiliated with a hospital	24 (48.0)
Diagnostic imaging center not affiliated with a hospital	7 (14.0)
Private practice/cardiology clinic/physician’s office	6 (12.0)
Region, n (%)	
Northeast	4 (8.0)
Midwest	12 (24.0)
West	28 (56.0)
South	6 (12.0)
Practice size, mean (SD)	
Nuclear technician/technologist	5.1 (5.0)
Nurse	6.5 (10.4)
Physician	15.7 (14.9)
Nurse practitioner/physician assistant	8.4 (11.9)
Cardiovascular technologist/assistant	2.8 (5.4)
Exercise physiologist	1.6 (3.7)
Other^b^	0.2 (0.4)
Weekly hours of operation, mean (SD)	43.9 (9.9)
Total cameras available for use in SPECT-MPI tests, mean (SD)	3.2 (3.3)
Number of patients that received SPECT-MPI using maximal exercise in the past 30 days, mean (SD)	134.2 (189.3)
Number of patients that received SPECT-MPI using PSA in the past 30 days, mean (SD)^c,d^	163.9 (230.4)
*de novo* stress tests	134.2 (206.9)
Inadequate EST converted to PSA	29.7 (43.7)
Use of low-level exercise along with PSA	16.9 (28.1)
Availability of protocol for transition to PSA, n (%)	
Yes	47 (94.0)
No	1 (2.0)
Don’t know/unsure	2 (4.0)
Decision makers for transition to PSA, n (%)^e^	
Nuclear technician/technologist	25 (50.0)
Nurse	34 (68.0)
Physician	20 (40.0)
Nurse practitioner/physician assistant	10 (20.0)
Cardiovascular technologist/assistant	9 (18.0)
Exercise physiologist	5 (10.0)

*EST*, exercise stress test; *PSA*, pharmacological stress agent; *SD*, standard deviation; *SPECT-MPI*, single-photon emission computed tomography myocardial perfusion imaging

^a^Other roles include president, supervisor, and test proctor

^b^Other staff type includes registrar

^c^Using regadenoson, adenosine, or dipyridamole

^d^Includes patients that received PSA with or without low-level exercise

^e^Respondents could select more than one staff type; data has been reported at the center level

Of the 50 centers represented in the survey, 35 (70%) used only regadenoson, 3 (6%) used only adenosine, 3 (6%) used regadenoson and adenosine, 7 (14%) used regadenoson and dipyridamole, and 2 (4%) used all 3 agents when converting patients from an inadequate EST to a PSA. The majority of the centers used regadenoson when converting patients from an inadequate EST to a PSA (n = 47 [94.0%], 8 [16.0%], and 9 [18.0%] for regadenoson, adenosine and dipyridamole, respectively). Among the reasons for using PSA in the past 30 days, 36 (76.6%) centers that used regadenoson reported convenience as the reason; additionally, 32 (68.1%) cited its reasonable adverse event profile, 5 (10.6%) needed regadenoson because other agents were contraindicated, and 3 (6.4%) used it due to its low cost. Comparatively, 6 (75.0%) centers that used adenosine reported low cost as the reason, and 2 (25.0%) centers each reported a reasonable adverse event profile and other (corporate policy, patient preference) reasons for its use. Among centers using dipyridamole, 4 (44.4%) centers each cited its low cost and contraindications to other agents, and 1 (11.1%) center each listed convenience, a reasonable adverse event profile, and other (physician preference) reasons.

### Factors Related to the Conversion of Patients

Factors related to the conversion of patients from an inadequate EST to a PSA are summarized in Table 2. Same-day conversion occurred for the majority of patients across agents (92%, 89%, and 98% for regadenoson, adenosine, and dipyridamole, respectively). The mean number of patients converted in the past 30 days was highest for regadenoson (n = 30), followed by adenosine (n = 9) and dipyridamole (n = 2), with most centers having the PSA readily available (ranging from 67% for dipyridamole to 98% for regadenoson). After an inadequate EST, mean patient wait time to receive the PSA was shortest for regadenoson (4.9 minutes vs 12.4 and 17.4 minutes for adenosine and dipyridamole, respectively). Centers using regadenoson (59.6%) following an inadequate EST were more likely to administer the PSA within a mean of 3 minutes during recovery, while only 1 center using dipyridamole reported administering the PSA within 3 minutes; no centers using adenosine reported administering the PSA within in a mean of 3 minutes.

**Table 2:** Factors Related to the Procedure/Practice to Convert Patients From Inadequate EST to PSA

Characteristic	Regadenoson	Adenosine	Dipyridamole
	N = 47	N = 8	N = 9
Factors related to transition to PSA			
Convenience of converting patients from inadequate EST to PSA, n (%)			
Very convenient	41 (87.2)	4 (50.0)	0 (0.0)
Somewhat convenient	3 (6.4)	2 (25.0)	5 (55.6)
Not convenient	3 (6.4)	2 (25.0)	4 (44.4)
Patients converted to each PSA in the past 30 days, mean (SD)	29.6 (44.1)	9.3 (9.9)	1.9 (1.8)
Patients converted on same day (%)	91.9	88.8	97.8
Laboratory scheduling impacted by same-day conversion from inadequate EST to PSA, n (%)			
Yes	10 (21.3)	1 (12.5)	3 (33.3)
No	37 (78.7)	6 (75.0)	5 (55.6)
Not applicable	0 (0.0)	1 (12.5)	1 (11.1)
PSA readily available, n (%)	46 (97.9)	7 (87.5)	6 (66.7)
Wait time before administration of PSA, mean (SD) (minutes)	4.9 (5.8)	12.4 (8.2)	17.4 (18.2)
Centers that administer the PSA within 3 minutes following an inadequate EST, n (%)	28 (59.6)	0 (0.0)	1 (11.1)
Factors related to administration of PSA			
Convenience of adjusting dosage by patient body weight/mass, n (%)			
Very convenient	N/A	3 (37.5)	3 (33.3)
Somewhat convenient	N/A	4 (50.0)	3 (33.3)
Not convenient	N/A	1 (12.5)	3 (33.3)
Complexity of administration, n (%)			
Extremely complex	0 (0.0)	0 (0.0)	1 (11.1)
Moderately complex	1 (2.1)	5 (62.5)	7 (77.8)
Not at all complex	46 (97.9)	3 (37.5)	1 (11.1)
Proportion of patients that experience adverse reactions, mean (SD)^a^	19.7 (22.4)	16.4 (18.9)	31.8 (25.1)

*EST*, exercise stress test; *PSA*, pharmacological stress agent; *SD*, standard deviation

^a^Adverse reactions that required some type of treatment or intervention

Centers were asked about the complexity of administering a PSA following an inadequate EST; how the levels of complexity were interpreted was left up to the discretion of the respondent. A large majority of centers rated regadenoson administration as “not at all complex” (98%), as opposed to 38% and 11% for adenosine and dipyridamole, respectively. Adenosine and dipyridamole administration were more commonly rated as “moderately complex” (63% and 78%, respectively). The proportions of patients experiencing adverse reactions were similar across the PSAs, at 20%, 16%, and 32% for regadenoson, adenosine, and dipyridamole, respectively (Table 2). Only adverse reactions requiring some type of treatment or intervention were reported. Of 12 centers that used >1 PSA, 11 centers (92%) found regadenoson to be the most preferred PSA (overall) among staff.

### Resource use and time

**Figure 1:**
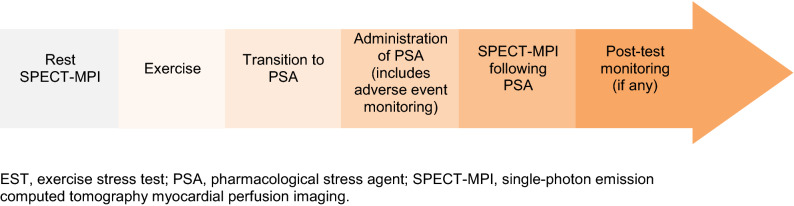
SPECT-MPI Patient Journey for Conversion From an Inadequate EST to a PSA. *EST*, exercise stress test; *PSA*, pharmacological stress agent; *SPECT-MPI*, single-photon emission computed tomography myocardial perfusion imaging

Total staff time (aggregating time for multiple staff members who may have worked concurrently at each step) required from PSA transition to post-test monitoring (Figure 1) was shortest for regadenoson (Table 3). The mean time needed in minutes for the transition to PSA was 10.5 minutes for regadenoson, 26.7 minutes for adenosine, and 36.9 minutes for dipyridamole. For administration, the mean time was 49.7 minutes for regadenoson, 83.5 minutes for adenosine, and 73.2 minutes for dipyridamole. Administration refers to the process from the start of PSA administration to the start of the SPECT-MPI procedure post-stress. This included, but was not limited to, PSA infusion time. For the remaining steps, the mean time was 41.5 minutes for regadenoson, 60.9 minutes for adenosine, and 53.0 minutes for dipyridamole for the SPECT-MPI following PSA and 8.8 minutes for regadenoson, 29.6 minutes for adenosine, and 15.9 minutes for dipyridamole for post-test monitoring. While the safety profile was comparable across agents, the total staff time involved with managing adverse reactions was shorter for regadenoson compared to adenosine or dipyridamole. Centers using regadenoson (34%) were less likely to conduct post-test monitoring, which included evaluating patient performance and managing adverse reactions, compared to 63% of centers using adenosine, and 44% of centers using dipyridamole. Staff numbers and time required for SPECT-MPI procedure with use of regadenoson compared to adenosine and dipyridamole, with a breakdown by staff role, are presented in Supplementary Tables 1 and 2, respectively.

**Table 3:** Resource use and time

	Mean (SD) total number of staff members per step	Mean (SD) total staff time^a^ in minutes per step
Transition to PSA		
Regadenoson	2.1 (1.0)	10.5 (1.0)
Adenosine	1.8 (1.2)	26.7 (2.5)
Dipyridamole	2.0 (1.0)	36.9 (3.9)
Administration of PSA^b^		
Regadenoson	2.9 (1.0)	49.7 (4.9)
Adenosine	3.0 (0.9)	83.5 (9.9)
Dipyridamole	2.6 (1.2)	73.2 (11.6)
SPECT-MPI following PSA		
Regadenoson	1.9 (1.0)	41.5 (9.8)
Adenosine	2.4 (1.2)	60.9 (15.7)
Dipyridamole	2.4 (1.1)	53.0 (9.5)
Post-test monitoring		
Regadenoson	0.7 (1.2)	8.8 (1.8)
Adenosine	1.6 (1.6)	29.6 (5.7)
Dipyridamole	0.8 (1.1)	15.9 (3.7)

*PSA*, pharmacological stress agent; *SD*, standard deviation; *SPECT-MPI*, single-photon emission computed tomography myocardial perfusion imaging

^a^Total staff time aggregates the time for multiple staff members who may have worked concurrently at each step

^b^Administration refers to the process from the start of PSA administration to the start of the SPECT-MPI procedure. This included but was not limited to PSA infusion time

## Discussion

Using an online survey, this study sought to characterize the resources, best practices, and time involved in PSA use during SPECT-MPI for patients with an inadequate EST from the perspective of nuclear imaging center staff. The findings show that regadenoson was the most commonly used and most preferred PSA for conversion after an inadequate EST. Regadenoson was used by centers because of convenience and a reasonable adverse event profile. The majority of patients using regadenoson were converted on the same day with minimal impact to laboratory scheduling. Note that while most centers in this study preferred regadenoson, other real-world studies with regadenoson as a comparator have demonstrated that adenosine is less expensive and results in fewer adverse events due to its short half-life; similar results have been seen for dipyridamole.^5^,^6^

Regadenoson administration was considered “not at all complex” by a majority of nuclear imaging center staff, who further benefited with respect to reductions in total staff time for each step of the conversion process relative to adenosine and dipyridamole. Compared to adenosine and dipyridamole, use of regadenoson required less nuclear imaging center staff time, which may be due to its convenient administration and rapid onset of action. As a time saving agent, regadenoson is associated with operational efficiency for nuclear imaging staff. This may also impact the overall costs to the center, and the experience of the patient during SPECT-MPI testing.

Observed benefits of regadenoson are consistent with and supplement findings from previous studies that have collectively demonstrated its potential to create more efficient/effective diagnosis for several reasons, including accurate stress testing, better use of patient time, and use of a more streamlined stress protocol.^7^–^11^ Friedman et al fielded an online survey to nuclear medicine technologists (n = 141) from cardiovascular imaging centers in the US to characterize regadenoson’s laboratory efficiency relative to adenosine and dipyridamole.^8^ Regadenoson was associated with the shortest total time for MPI testing, as well as time from PSA administration to the start of imaging and to adverse event management, thereby offering the most operational efficiency. Another study described higher satisfaction with regadenoson vs dipyridamole from both clinician/technologist and patient standpoints when using validated questionnaires.^9^ Most recently, the aforementioned EXERRT trial demonstrated the efficacy and safety of administering regadenoson at 3 minutes of recovery after an inadequate EST, provided that patients do not present with electrocardiographic changes or other signs or symptoms of ischemia.^5^ Of note, the survey found that a majority of centers using regadenoson administered regadenoson within 3 minutes on average for patients experiencing an inadequate EST.

We acknowledge the limitations of this study. First, obtaining data via an online survey may limit the generalizability of the findings. For example, the nuclear imaging center staff who agreed to participate may have provided responses that are not representative of all US nuclear imaging centers that perform SPECT-MPI using PSAs after inadequate ESTs. Roles varied among survey respondents, and some groups were more represented (e.g., nuclear technicians) than others (e.g., physicians). As a result, the findings may not be broadly generalizable to the less represented roles. Surveying nuclear lab directors only may have produced more generalizable results considering the purpose of this study was to assess resource use in the nuclear lab setting. Regadenoson use was highly represented among survey respondents, while a smaller number of centers that reported using adenosine or dipyridamole responded to the survey. For example, one would expect that adenosine’s post-monitoring period would be comparatively short due to the short half-life of the agent. However, the small sample size (only 3 sites collected data for adenosine monitoring time) prevented us from drawing conclusions regarding this counter-intuitive observation. Given this, results associated with adenosine and dipyridamole use should be interpreted carefully, as trends could differ with a larger sample. Additionally, there may be other factors related to the efficiency of PSAs during SPECT-MPI that were not specified in the survey.

In conclusion, regadenoson is preferred by nuclear imaging center staff and associated with operational efficiency for converting patients from inadequate EST to PSA in real-world practice.

## New Knowledge Gained

This article provides new information on the efficiency of PSA use for patients converting from EST to PSA SPECT-MPI tests in real-world settings and confirms same-day conversion occurs for the majority of patients. Past research on the testing of conversion patients has been in clinical trial settings, focused on a subset of the available stress agents on the market, or been limited to perspectives from staff at single centers. This study compares real-world use of adenosine, dipyridamole, and regadenoson across 50 imaging centers in the US, including a breakdown of staff time requirements at each step of the testing process.

## Supplementary Information

Below is the link to the electronic supplementary material.

Electronic supplementary material 1 (PPTX 163 kb)

Electronic supplementary material 2 (PDF 52 kb)

Electronic supplementary material 3 (PDF 73 kb)

## Abbreviations

EST: Exercise stress test
PSA: Pharmacological stress agent
SPECT-MPI: Single-photon emission computed tomography myocardial perfusion imaging
