# Supplementary material for: Evaluating the use of pharmacological stress agents during single-photon emission computed tomography myocardial perfusion imaging tests after inadequate exercise stress test
Source: J Nucl Cardiol. 2021 Mar 11;29(4):1788–95. doi: 10.1007/s12350-021-02546-5 (PMC9345818; doi:10.1007/s12350-021-02546-5)
Supplement: Supplementary file 1 — Electronic supplementary material 1 (PPTX 163 kb) [file 12350_2021_2546_MOESM1_ESM.pptx]

## Slide 1
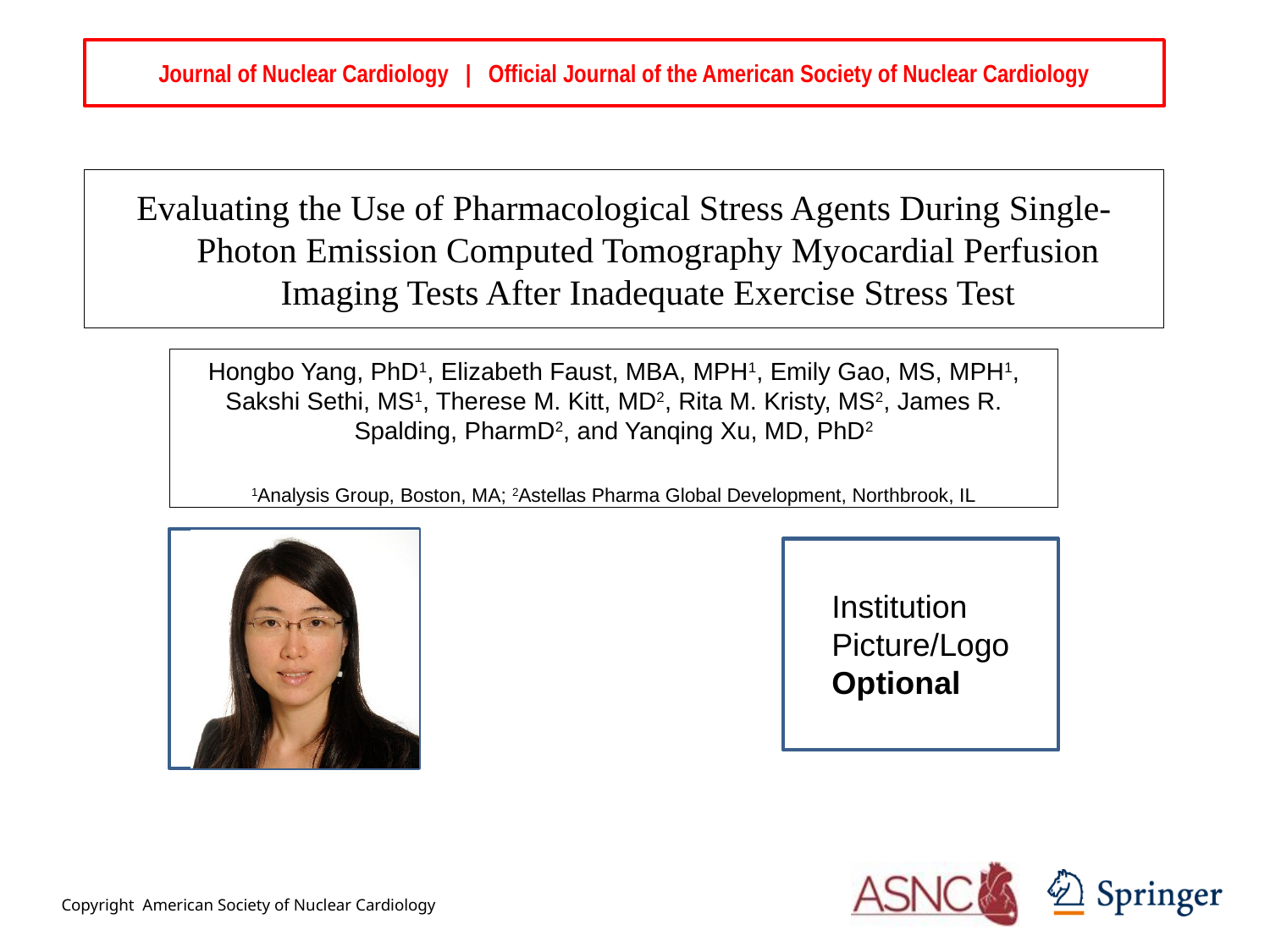

Journal of Nuclear Cardiology | Official Journal of the American Society of Nuclear Cardiology
# Evaluating the Use of Pharmacological Stress Agents During Single-Photon Emission Computed Tomography Myocardial Perfusion Imaging Tests After Inadequate Exercise Stress Test
Hongbo Yang, PhD1, Elizabeth Faust, MBA, MPH1, Emily Gao, MS, MPH1, Sakshi Sethi, MS1, Therese M. Kitt, MD2, Rita M. Kristy, MS2, James R. Spalding, PharmD2, and Yanqing Xu, MD, PhD2
1Analysis Group, Boston, MA; 2Astellas Pharma Global Development, Northbrook, IL
Head shot of author
required
Institution
Picture/Logo
Optional
Copyright American Society of Nuclear Cardiology

## Slide 2
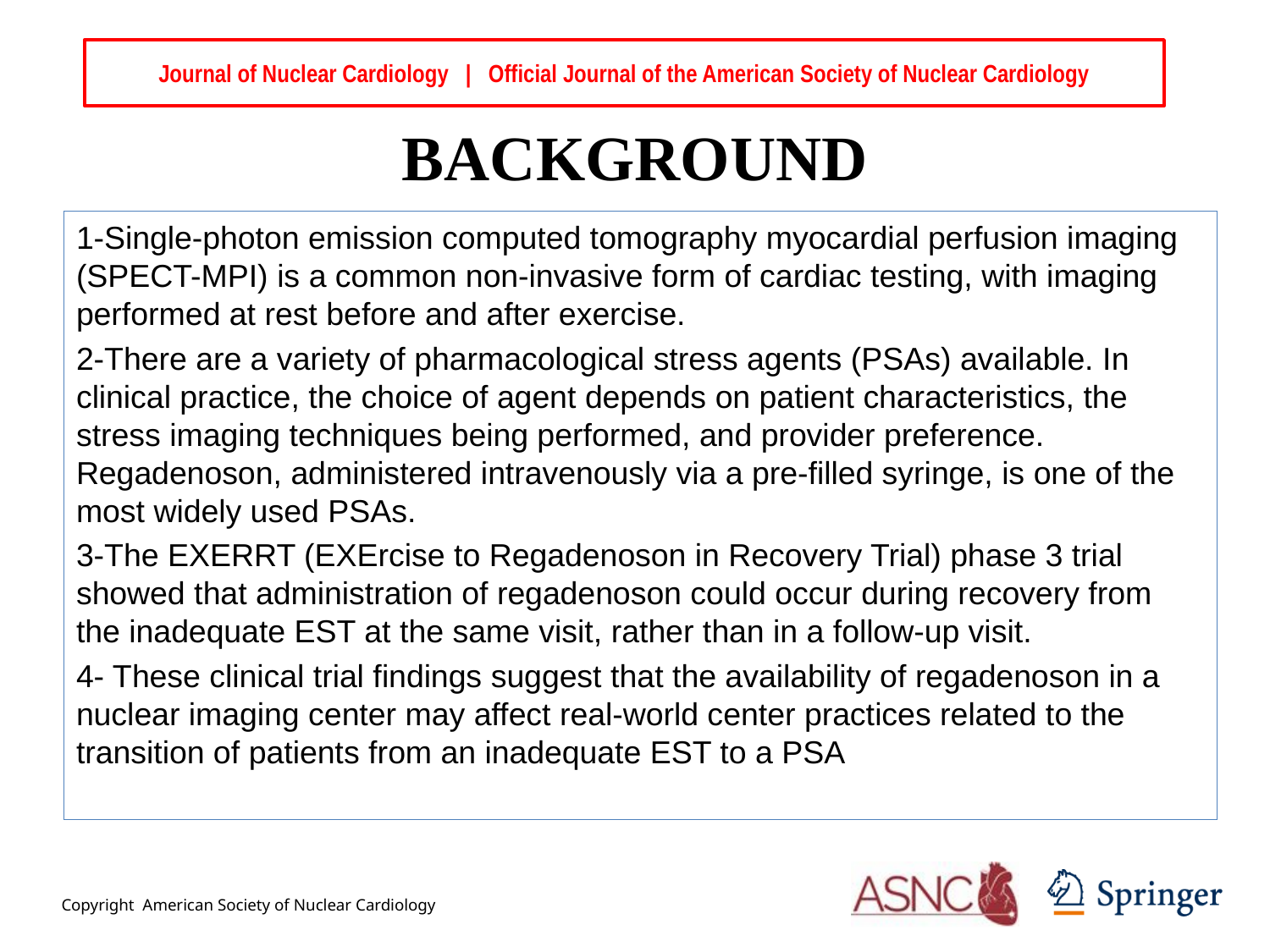

Journal of Nuclear Cardiology | Official Journal of the American Society of Nuclear Cardiology
# BACKGROUND
1-Single-photon emission computed tomography myocardial perfusion imaging (SPECT-MPI) is a common non-invasive form of cardiac testing, with imaging performed at rest before and after exercise.
2-There are a variety of pharmacological stress agents (PSAs) available. In clinical practice, the choice of agent depends on patient characteristics, the stress imaging techniques being performed, and provider preference. Regadenoson, administered intravenously via a pre-filled syringe, is one of the most widely used PSAs.
3-The EXERRT (EXErcise to Regadenoson in Recovery Trial) phase 3 trial showed that administration of regadenoson could occur during recovery from the inadequate EST at the same visit, rather than in a follow-up visit.
4- These clinical trial findings suggest that the availability of regadenoson in a nuclear imaging center may affect real-world center practices related to the transition of patients from an inadequate EST to a PSA
Copyright American Society of Nuclear Cardiology

## Slide 3
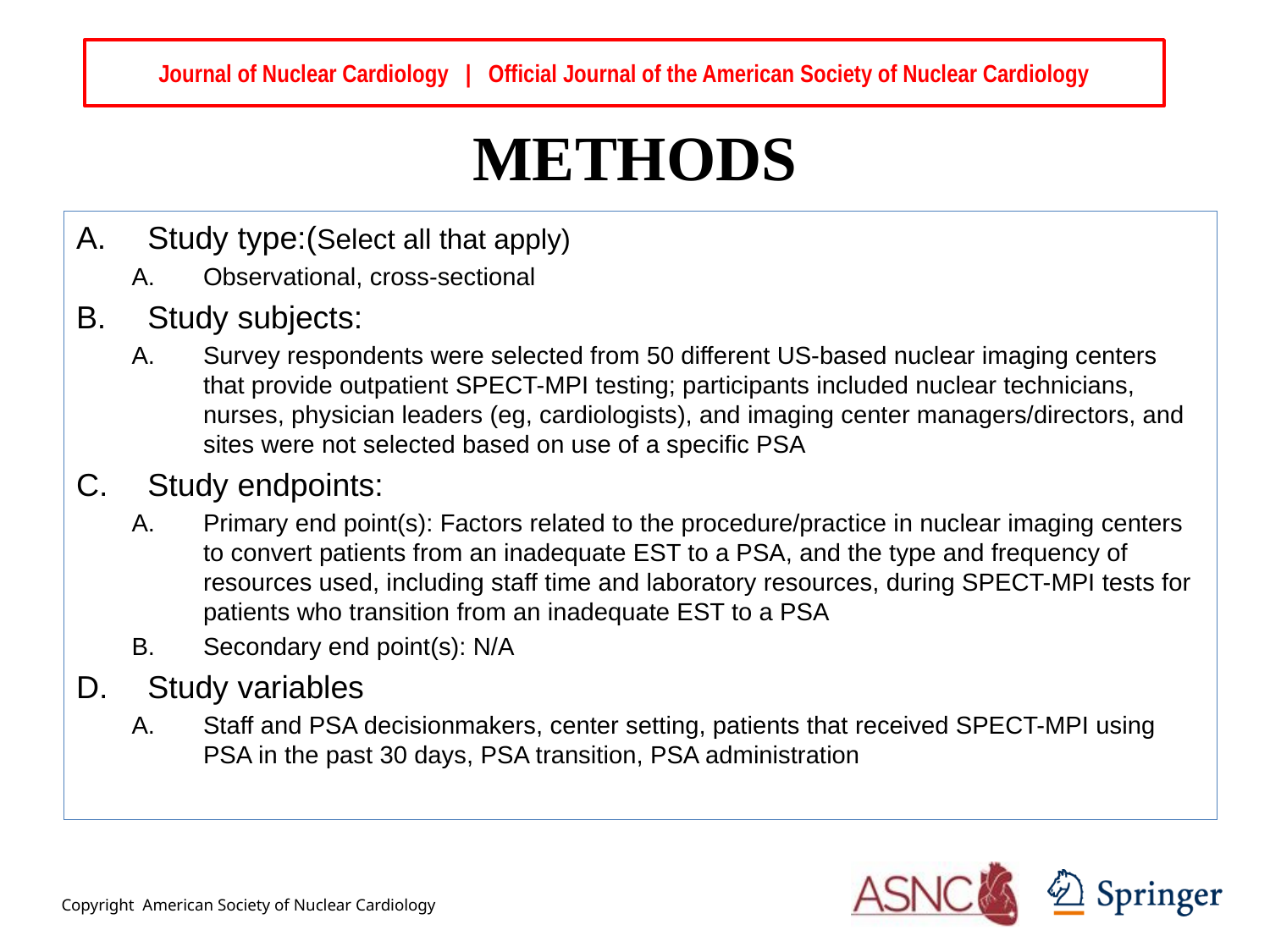

Journal of Nuclear Cardiology | Official Journal of the American Society of Nuclear Cardiology
# METHODS
Study type:(Select all that apply)
Observational, cross-sectional
Study subjects:
Survey respondents were selected from 50 different US-based nuclear imaging centers that provide outpatient SPECT-MPI testing; participants included nuclear technicians, nurses, physician leaders (eg, cardiologists), and imaging center managers/directors, and sites were not selected based on use of a specific PSA
Study endpoints:
Primary end point(s): Factors related to the procedure/practice in nuclear imaging centers to convert patients from an inadequate EST to a PSA, and the type and frequency of resources used, including staff time and laboratory resources, during SPECT-MPI tests for patients who transition from an inadequate EST to a PSA
Secondary end point(s): N/A
Study variables
Staff and PSA decisionmakers, center setting, patients that received SPECT-MPI using PSA in the past 30 days, PSA transition, PSA administration
Copyright American Society of Nuclear Cardiology

## Slide 4
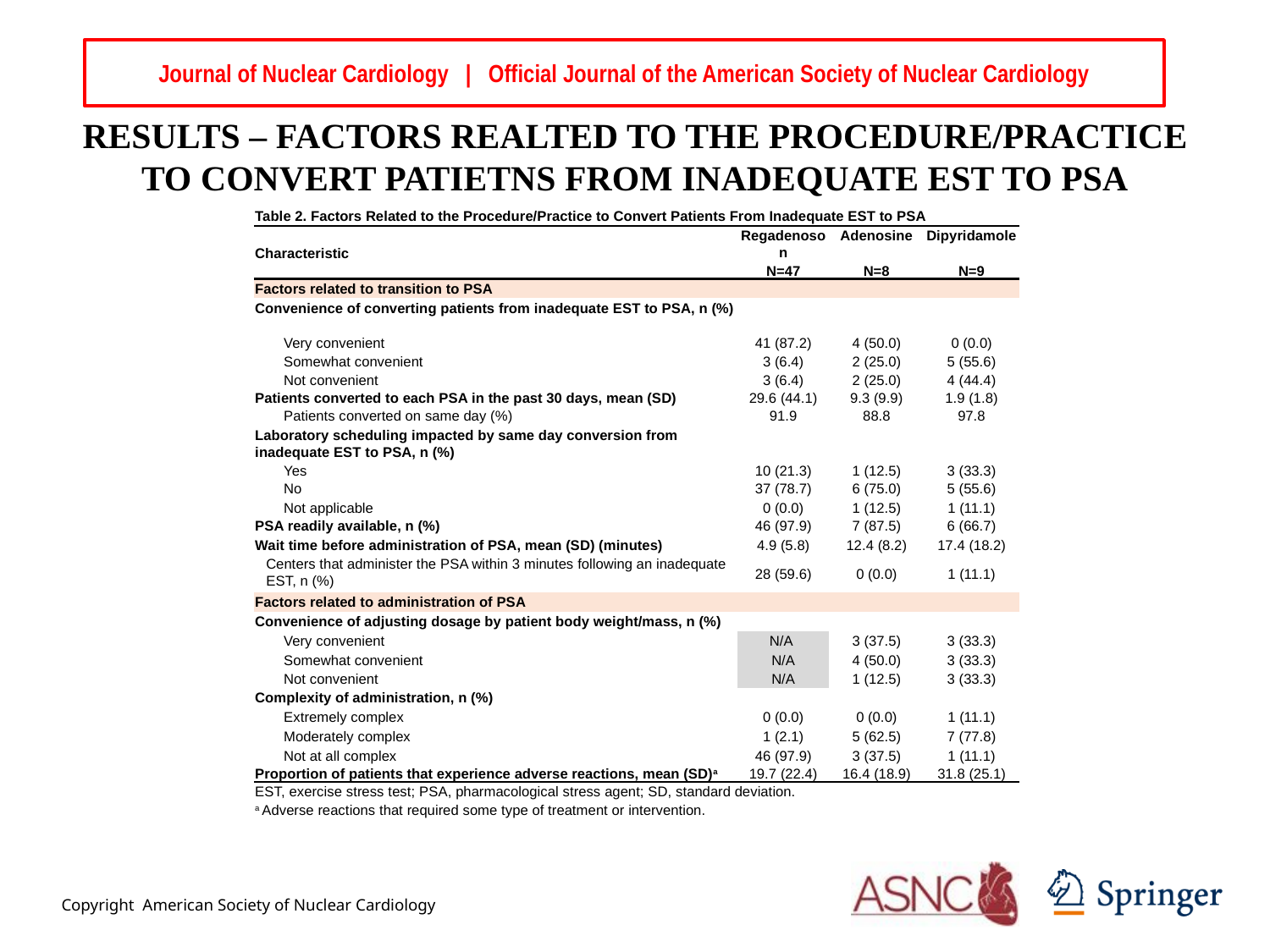

Journal of Nuclear Cardiology | Official Journal of the American Society of Nuclear Cardiology
# RESULTS – FACTORS REALTED TO THE PROCEDURE/PRACTICE TO CONVERT PATIETNS FROM INADEQUATE EST TO PSA
| Table 2. Factors Related to the Procedure/Practice to Convert Patients From Inadequate EST to PSA | | | |
| --- | --- | --- | --- |
| Characteristic | Regadenoson | Adenosine | Dipyridamole |
| | N=47 | N=8 | N=9 |
| Factors related to transition to PSA | | | |
| Convenience of converting patients from inadequate EST to PSA, n (%) | | | |
| Very convenient | 41 (87.2) | 4 (50.0) | 0 (0.0) |
| Somewhat convenient | 3 (6.4) | 2 (25.0) | 5 (55.6) |
| Not convenient | 3 (6.4) | 2 (25.0) | 4 (44.4) |
| Patients converted to each PSA in the past 30 days, mean (SD) | 29.6 (44.1) | 9.3 (9.9) | 1.9 (1.8) |
| Patients converted on same day (%) | 91.9 | 88.8 | 97.8 |
| Laboratory scheduling impacted by same day conversion from inadequate EST to PSA, n (%) | | | |
| Yes | 10 (21.3) | 1 (12.5) | 3 (33.3) |
| No | 37 (78.7) | 6 (75.0) | 5 (55.6) |
| Not applicable | 0 (0.0) | 1 (12.5) | 1 (11.1) |
| PSA readily available, n (%) | 46 (97.9) | 7 (87.5) | 6 (66.7) |
| Wait time before administration of PSA, mean (SD) (minutes) | 4.9 (5.8) | 12.4 (8.2) | 17.4 (18.2) |
| Centers that administer the PSA within 3 minutes following an inadequate EST, n (%) | 28 (59.6) | 0 (0.0) | 1 (11.1) |
| Factors related to administration of PSA | | | |
| Convenience of adjusting dosage by patient body weight/mass, n (%) | | | |
| Very convenient | N/A | 3 (37.5) | 3 (33.3) |
| Somewhat convenient | N/A | 4 (50.0) | 3 (33.3) |
| Not convenient | N/A | 1 (12.5) | 3 (33.3) |
| Complexity of administration, n (%) | | | |
| Extremely complex | 0 (0.0) | 0 (0.0) | 1 (11.1) |
| Moderately complex | 1 (2.1) | 5 (62.5) | 7 (77.8) |
| Not at all complex | 46 (97.9) | 3 (37.5) | 1 (11.1) |
| Proportion of patients that experience adverse reactions, mean (SD)a | 19.7 (22.4) | 16.4 (18.9) | 31.8 (25.1) |
| EST, exercise stress test; PSA, pharmacological stress agent; SD, standard deviation. | | | |
| a Adverse reactions that required some type of treatment or intervention. | | | |
Copyright American Society of Nuclear Cardiology

## Slide 5
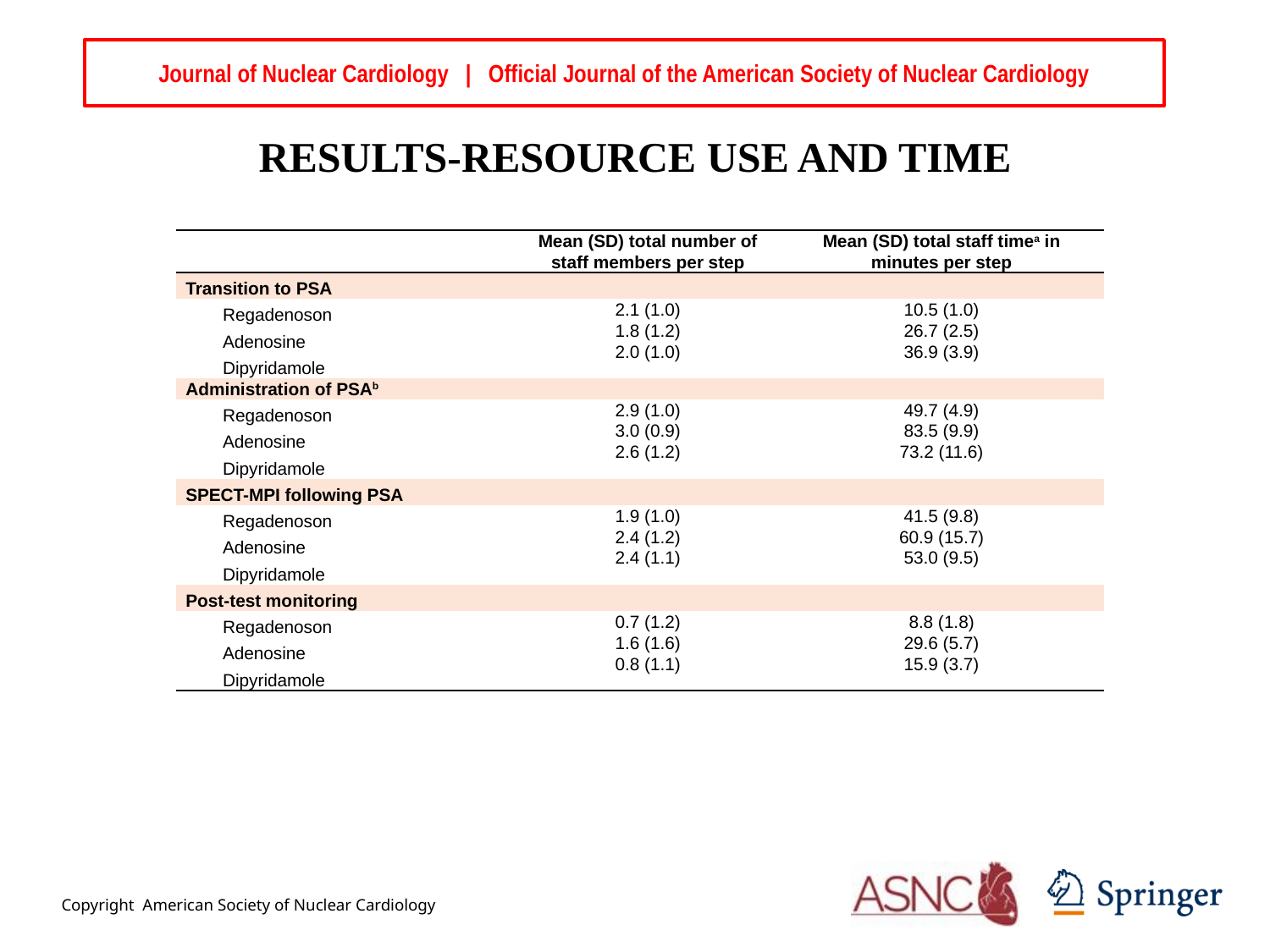

Journal of Nuclear Cardiology | Official Journal of the American Society of Nuclear Cardiology
# RESULTS-RESOURCE USE AND TIME
| | Mean (SD) total number of staff members per step | Mean (SD) total staff timea in minutes per step |
| --- | --- | --- |
| Transition to PSA | | |
| Regadenoson Adenosine Dipyridamole | 2.1 (1.0) 1.8 (1.2) 2.0 (1.0) | 10.5 (1.0) 26.7 (2.5) 36.9 (3.9) |
| Administration of PSAb | | |
| Regadenoson Adenosine Dipyridamole | 2.9 (1.0) 3.0 (0.9) 2.6 (1.2) | 49.7 (4.9) 83.5 (9.9) 73.2 (11.6) |
| SPECT-MPI following PSA | | |
| Regadenoson Adenosine Dipyridamole | 1.9 (1.0) 2.4 (1.2) 2.4 (1.1) | 41.5 (9.8) 60.9 (15.7) 53.0 (9.5) |
| Post-test monitoring | | |
| Regadenoson Adenosine Dipyridamole | 0.7 (1.2) 1.6 (1.6) 0.8 (1.1) | 8.8 (1.8) 29.6 (5.7) 15.9 (3.7) |
Copyright American Society of Nuclear Cardiology

## Slide 6
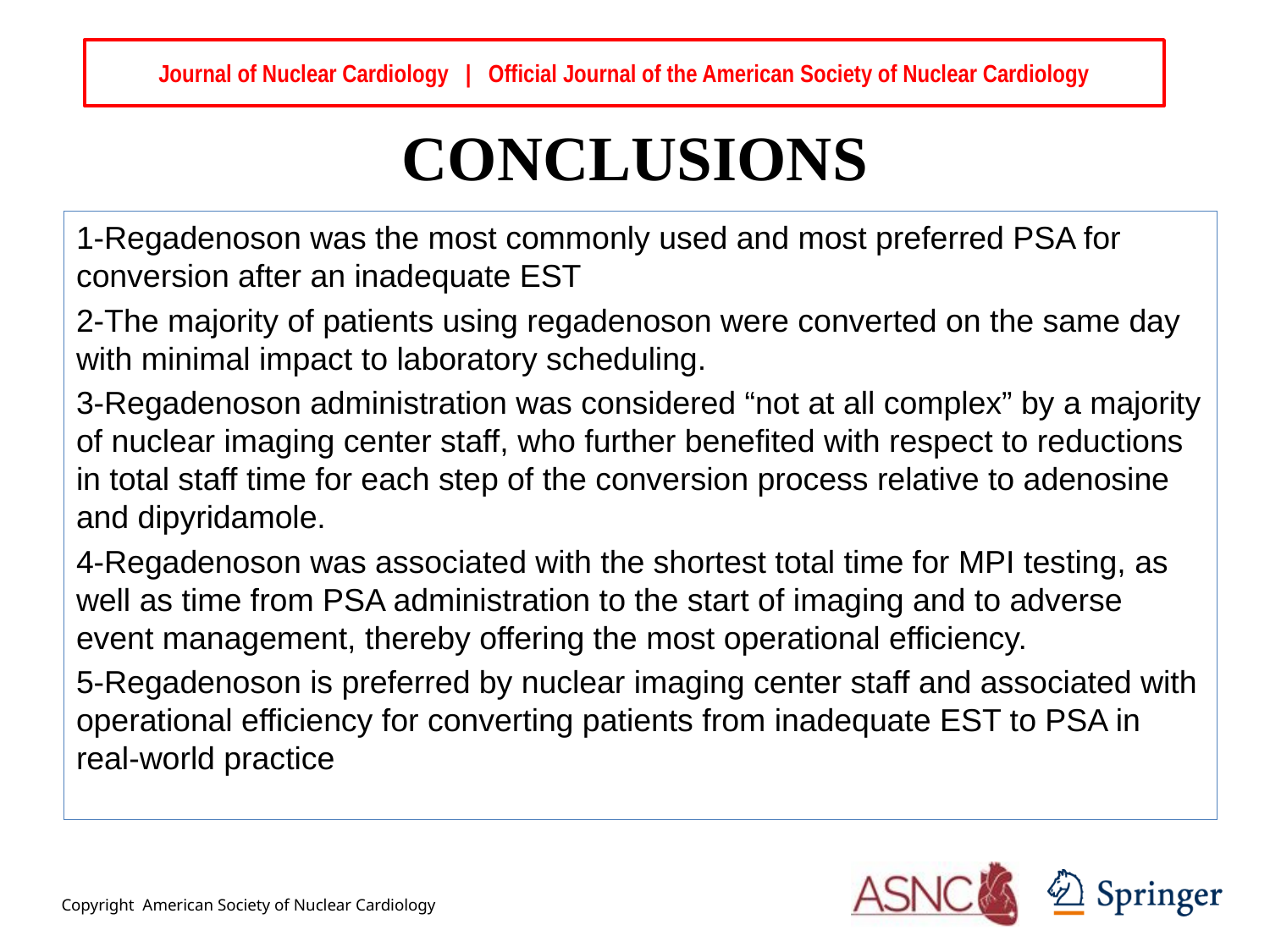

Journal of Nuclear Cardiology | Official Journal of the American Society of Nuclear Cardiology
# CONCLUSIONS
1-Regadenoson was the most commonly used and most preferred PSA for conversion after an inadequate EST
2-The majority of patients using regadenoson were converted on the same day with minimal impact to laboratory scheduling.
3-Regadenoson administration was considered “not at all complex” by a majority of nuclear imaging center staff, who further benefited with respect to reductions in total staff time for each step of the conversion process relative to adenosine and dipyridamole.
4-Regadenoson was associated with the shortest total time for MPI testing, as well as time from PSA administration to the start of imaging and to adverse event management, thereby offering the most operational efficiency.
5-Regadenoson is preferred by nuclear imaging center staff and associated with operational efficiency for converting patients from inadequate EST to PSA in real-world practice
Copyright American Society of Nuclear Cardiology
