# Supplementary material for: Evaluating the use of pharmacological stress agents during single-photon emission computed tomography myocardial perfusion imaging tests after inadequate exercise stress test
Source: J Nucl Cardiol. 2021 Mar 11;29(4):1788–95. doi: 10.1007/s12350-021-02546-5 (PMC9345818; doi:10.1007/s12350-021-02546-5)
Supplement: Supplementary file 2 — Electronic supplementary material 2 (PDF 52 kb) [file 12350_2021_2546_MOESM2_ESM.pdf]

Supplemental Table 1. Number of Staff Required for SPECT-MPI Procedure (Breakdown by Staff Role)

SPECT-MPI steps	Nuclear technician / technologist	Nurse	Physician	Nurse practitioner / physician assistant	Cardiovascular technologist / assistant	Exercise physiologist
	Mean (SD)	Mean (SD)	Mean (SD)	Mean (SD)	Mean (SD)	Mean (SD)
**1. Rest SPECT-MPI**	1.3 (0.5)	0.4 (0.6)	0.3 (0.5)	0.1 (0.3)	0.2 (0.4)	0.1 (0.3)
**2. Exercise**	1.0 (0.3)	0.8 (0.5)	0.5 (0.5)	0.3 (0.5)	0.4 (0.5)	0.2 (0.4)
**3. Transition to PSA[a]**						
Regadenoson (N=47)	0.5 (0.5)	0.7 (0.5)	0.4 (0.5)	0.2 (0.4)	0.2 (0.4)	0.1 (0.3)
Adenosine (N=8)	0.4 (0.5)	0.4 (0.5)	0.3 (0.5)	0.4 (0.5)	0.4 (0.5)	0.0 (0.0)
Dipyridamole (N=9)	0.2 (0.4)	0.8 (0.4)	0.6 (0.5)	0.2 (0.7)	0.1 (0.3)	0.1 (0.3)
**4. Administration of PSA**						
Regadenoson (N=47)	0.9 (0.2)	0.8 (0.5)	0.5 (0.5)	0.2 (0.5)	0.3 (0.5)	0.1 (0.4)
Adenosine (N=8)	1.0 (0.5)	0.8 (0.7)	0.4 (0.5)	0.4 (0.5)	0.5 (0.5)	0.0 (0.0)
Dipyridamole (N=9)	0.7 (0.5)	1.0 (0.5)	0.6 (0.5)	0.0 (0.0)	0.2 (0.4)	0.1 (0.3)
**4a. Managing adverse reactions**						
Regadenoson (N=47)	0.6 (0.5)	0.8 (0.5)	0.6 (0.5)	0.3 (0.5)	0.3 (0.4)	0.1 (0.4)
Adenosine (N=8)	0.5 (0.5)	0.8 (0.7)	0.4 (0.5)	0.4 (0.5)	0.4 (0.5)	0.0 (0.0)
Dipyridamole (N=9)	0.6 (0.5)	1.0 (0.7)	0.6 (0.5)	0.0 (0.0)	0.0 (0.0)	0.1 (0.3)
**5. SPECT-MPI following PSA**						
Regadenoson (N=47)	1.0 (0.3)	0.4 (0.5)	0.2 (0.4)	0.1 (0.2)	0.1 (0.3)	0.1 (0.3)
Adenosine (N=8)	1.1 (0.6)	0.5 (0.8)	0.1 (0.4)	0.4 (0.5)	0.3 (0.5)	0.0 (0.0)
Dipyridamole (N=9)	0.9 (0.3)	0.8 (0.7)	0.6 (0.5)	0.0 (0.0)	0.1 (0.3)	0.1 (0.3)
**6. Post-test monitoring**						
Regadenoson (N=47)	0.3 (0.5)	0.2 (0.5)	0.0 (0.2)	0.0 (0.2)	0.1 (0.3)	0.0 (0.2)
Adenosine (N=8)	0.5 (0.8)	0.5 (0.8)	0.0 (0.0)	0.3 (0.5)	0.4 (0.5)	0.0 (0.0)
Dipyridamole (N=9)	0.2 (0.4)	0.4 (0.7)	0.0 (0.0)	0.0 (0.0)	0.1 (0.3)	0.0 (0.0)

PSA, pharmacological stress agent; SD, standard deviation; SPECT-MPI, single-photon emission computed tomography myocardial perfusion imaging.

[a] Center level data has been presented for centers that use each PSA.
