# Supplementary material for: Evaluating the use of pharmacological stress agents during single-photon emission computed tomography myocardial perfusion imaging tests after inadequate exercise stress test
Source: J Nucl Cardiol. 2021 Mar 11;29(4):1788–95. doi: 10.1007/s12350-021-02546-5 (PMC9345818; doi:10.1007/s12350-021-02546-5)
Supplement: Supplementary file 3 — Electronic supplementary material 3 (PDF 73 kb) [file 12350_2021_2546_MOESM3_ESM.pdf]

Supplemental Table 2. Staff Time Required for SPECT-MPI Procedure (Minutes) (Breakdown by Staff Role)

SPECT-MPI steps[a]	Nuclear technician / technologist	Nurse	Physician	Nurse practitioner / Physician assistant	Cardiovascular technologist / assistant	Exercise physiologist
	Mean (SD)	Mean (SD)	Mean (SD)	Mean (SD)	Mean (SD)	Mean (SD)
**1. Rest SPECT-MPI**	43.1 (27.6)	23.9 (12.5)	29.0 (25.1)	27.5 (12.6)	25.0 (15.6)	33.8 (18.9)
**2. Exercise**	28.1 (25.6)	25.1 (10.1)	21.0 (15.4)	21.4 (11.6)	26.5 (7.3)	30.6 (11.0)
**3. Transition to PSA[b]**						
Regadenoson (N=47)	5.2 (6.3)	4.0 (4.8)	5.3 (6.3)	8.0 (8.8)	4.5 (7.0)	5.4 (8.2)
Adenosine (N=8)	18.3 (7.6)	11.7 (11.5)	15.0 (7.1)	13.0 (10.8)	18.3 (7.6)	—
Dipyridamole (N=9)	20.0 (14.1)	12.3 (8.5)	20.2 (23.0)	20.0 (—)	5.0 (—)	60.0 (—)
**4. Administration of PSA[c]**						
Regadenoson (N=47)	14.1 (15.3)	19.3 (11.8)	16.4 (13.1)	15.6 (9.0)	18.9 (7.4)	23.9 (12.1)
Adenosine (N=8)	29.1 (41.0)	25.0 (21.8)	30.0 (26.5)	21.7 (7.6)	32.5 (10.4)	—
Dipyridamole (N=9)	23.0 (8.8)	30.6 (15.5)	34.0 (15.2)	—	22.5 (10.6)	30.0 (—)
**4a. Managing adverse reactions**						
Regadenoson (N=47)	10.2 (9.4)	14.5 (9.8)	8.6 (6.9)	11.6 (6.5)	16.3 (7.7)	12.0 (7.4)
Adenosine (N=8)	21.3 (16.5)	20.0 (11.7)	21.7 (16.1)	13.3 (10.4)	20.0 (13.2)	—
Dipyridamole (N=9)	11.4 (18.9)	25.7 (12.7)	17.2 (19.3)	—	—	20.0 (—)
**5. SPECT-MPI following PSA**						
Regadenoson (N=47)	26.2 (15.9)	19.4 (17.0)	14.9 (21.8)	5.7 (8.1)	8.8 (4.8)	32.5 (8.7)
Adenosine (N=8)	37.1 (28.6)	16.7 (2.9)	20.0 (—)	13.7 (10.0)	12.5 (3.5)	—
Dipyridamole (N=9)	25.0 (10.7)	24.2 (14.6)	13.6 (15.0)	—	10.0 (—)	30.0 (—)
**6. Post-test monitoring**						
Regadenoson (N=47)	19.2 (12.3)	10.0 (5.5)	1.0 (0.0)	8.0 (2.8)	9.2 (1.8)	10.5 (6.4)
Adenosine (N=8)	30.0 (26.5)	15.0 (5.0)	—	10.5 (13.4)	12.0 (9.8)	—
Dipyridamole (N=9)	30.0 (0.0)	18.3 (10.4)	—	—	10.0 (—)	—

PSA, pharmacological stress agent; SD, standard deviation; SPECT-MPI, single-photon emission computed tomography myocardial perfusion imaging.

[a] Staff time required has been presented for staff types that were involved in each SPECT-MPI step.

[b] Staff time for transition to PSA is based on time to obtain/prepare PSA and wait time prior to administration of PSA.

[c] Administration refers to the process from the start of PSA administration to the start of the SPECT-MPI procedure. This included but was not limited to PSA infusion time.
